# The Antifungal Efficacy of Pure Garlic, Onion, and Lemon Extracts Against Candida albicans

**DOI:** 10.7759/cureus.38637

**Published:** 2023-05-06

**Authors:** Rita M Khounganian, Abdulaziz Alwakeel, Abdulhakim Albadah, Abdulrahman Nakshabandi, Shahad Alharbi, Ahmed S Almslam

**Affiliations:** 1 Oral Medicine and Diagnostic Sciences, College of Dentistry, King Saud University, Riyadh, SAU; 2 Oral Medicine and Oral Pathology, Tabuk Dental Center, Tabuk, SAU; 3 Microbiology Laboratory, College of Dentistry, King Saud University, Riyadh, SAU; 4 Oral Medicine, Harvard University, Cambridge, USA; 5 Oral Medicine and Oral Pathology, Buraidah Central Hospital, Buraydah, SAU; 6 Dentistry, College of Dentistry, King Saud University, Riyadh, SAU

**Keywords:** antifungal efficacy, lemon juice extract, onion, garlic, candida albicans

## Abstract

Introduction: The oral cavity is considered to be one of the most intricate environments in the human body. It is known to harbor commensal microorganisms that do not cause diseases, such as *Candida albicans*, a yeast fungus that has a carriage rate that tends to increase with age. It is worth noting that *C. albicans *can be readily identified within the flora of the gastrointestinal tract in 80% of healthy patients. Traditional medicine has alternatively been shown to play a key role in various health amenities with a wide spectrum anti-microbial effect against various yeast molds.

Objectives: To evaluate the antifungal efficacy of pure garlic, onion, and lemon juice extracts against *C. albicans. *

Materials and methods: *C. albicans* (ATCC 10231) were sub-cultured in brain agar followed by anaerobic incubation for 48 hours at 37°C. Ten plates were used for each of the materials studied to evaluate their antifungal efficacy against *C. albicans.* The efficiency of commercially available fresh garlic, onion, and lemon was tested in isolation against *C. albicans. *One-way ANOVA and chi-square were used for comparison between the different materials. The inhibition zone was measured, and the level of statistical significance was set at ≤0.05.

Results: The diameter of inhibition zones has been measured along the vertical and horizontal axis. No inhibition zones were observed for the onion and lemon extracts used in this study whereas the garlic extract exhibited inhibition zones with altered sizes (4.89 ± 0.275). A highly significant difference was observed between groups (P = 0.000) and between garlic and the other materials (P = 0.000).

Conclusions: Pure garlic showed a highly significant antifungal efficacy when compared to the onion and lemon juice extracts against *C*.* albicans*. Further studies are needed using different concentrations of onion, lemon, and lemon peel juice to confirm their antifungal efficacy in addition to their actual antimicrobial benefits.

## Introduction

The oral cavity is considered to be one of the most intricate environments in the human body. It is known to harbor commensal microorganisms that do not cause disease [1]. Of these commensal microorganisms is *Candida albicans*, a yeast fungus that has a carriage rate that tends to increase with age [2]. It is worth noting that, *C. albicans* can be readily identified within the flora of the gastrointestinal tract in 80% of healthy patients [3]. Recent studies have shown an alarming increase in the incidence of oral candidiasis, a customary oral fungal infection caused by *C. albicans*.

The incidence of candidiasis in the oral cavity with predominant *C. albicans* isolation has been reported to be 45% in neonates [4], 45-65% in children [5], 30-45% in healthy adults [6], 50-65% in cases of long-term denture wearers [7], 65-88% in those residing in acute and long-term facilities [8-10], 90% in patients with acute leukemia undergoing chemotherapy [11], and 95% in patients with HIV infection [12]. Current understanding regarding the behavior of *C. albicans* has also established the significant role of the microorganism in numerous oral and precancerous lesions [13]. That being said, this noticeable increase in the prevalence and incidence of the opportunistic pathogen may be attributed to the increasing number of immunocompromised patients [14]. Fungal infections are specifically an important cause of morbidity and mortality in immunosuppressed people [15].

With the subtle nature of *C. albicans*, little is still known regarding its invasive behavior; on the other hand, a few factors are believed to be of importance such as the protein-protein interaction that takes place between *Candida* cell walls and host cells, in addition to the ability of *Candida* to translocate through the epithelial mucosa [16].

Primarily, there are four major classes of antifungal medications that are utilized in the treatment of invasive fungal infections, including polyenes, pyrimidine analogs, echinocandins, and triazoles [17]. Unfortunately, the archaic prescription of antifungal medications has led to the emergence of fungal pathogens that have developed resistance to commonly used antifungals [17,18].

Traditional medicine has alternatively been shown to play a key role in various health amenities. Aromatic essential oils such as olive oil and cinnamon oil have been shown to be potent agents with a wide spectrum anti-microbial effect against various yeast molds [19].

The effect of fresh garlic (*Allium sativum* L) extract against *Candida* has been presented in vitro and has been ascribed to the action of allicin [20], a sulfur-containing compound that is formed at levels of approximately 3-5 mg/g of freshly crushed or sliced garlic cloves [21], which was exhibited by previous investigators to impede the proliferation of bacteria and fungi [3,22]. Allicin has the capability to shield against the overgrowth of *C. albicans* and diminish the risk of candida attaching itself to the cells lining the oral cavity [23]. While lemons carry some anti-fungal properties, they primarily work by detoxing the liver as it fights off *Candida* [19]. Citrus lemon peel contains terpenoids that have the capability of restraining the synthesis of ergosterol, which is a constituent of the fungal cell wall that plays a role in the maintenance of the permeability of the cell membrane. The essential oils extracted from the citrus lemon peel are anticipated to inhibit the growth of *C. albicans* [24], whereas onion (*Allium cepa* L) is a well-known nutraceutical and medicinal plant that is cultivated and used all over the globe [25]. It has countless health implications and is consumed for its alleged nutritional and health benefits for centuries [22]. Onion is a good natural source of flavonoids, specifically flavonols-quercetin and kaempferol, which are present as their glycosides [26].

Shon et al. [27] have pointed out the beneficial effects of flavonoids as antiallergenic, anti-inflammatory, anti-carcinogenic, and antioxidant properties. Consequentially, the food industry is interested in developing natural components for the total or partial replacement of synthetic antimicrobials [28].

## Materials and methods

Fungal strains and culture conditions

*C. albicans* (ATCC 10231) fungal-type culture collection was sub-cultured in brain agar followed by anaerobic incubation for 48 hours at 37°C. Ten plates were used for each of the materials studied to evaluate their antifungal efficacy against *C. albicans*.

Preparation of solutions

Commercially available fresh garlic, onion, and lemon were included in the present study. Their efficiency was tested in isolation against *C. albicans*. Garlic and onion were separately crushed into a paste using a sterilized mortar and pestle and kept in a sterile test tube until use. The surface of the lemon was disinfected with plain water at first and the extract was collected by cutting the lemon into two pieces and squeezing the secretion into a sterile beaker. Concentrations (100%) of all the prepared sterile extracts were placed in sterile discs and accordingly used within the present research.

To test the antifungal efficacy of the garlic, onion, and lemon extracts against *C. albicans*, the well-diffusion method was utilized for assessment by measuring the zone of inhibition around the well (vertical and horizontal line) using brain agar and Mueller Hinton agar plates. The colonies were adjusted according to the McFarland standard (0.5) and were swabbed onto the plates. Using a cork borer, 8 mm wells were created. A total of 50 μl of freshly prepared extracts of each material were pipetted using a micropipette and accordingly added to the wells followed by anaerobic incubation of the plates for 24 to 48 hours using the anaerobic jar, and the zone around the wells was measured.

The antifungal activity of the garlic, onion, and lemon extracts was independently carried out and assessed by considering the triplicates of all the extracts. Based on the acquired results, the combination effect of the performed extracts was analyzed.

Statistical analysis

One-way ANOVA and chi-square were used for comparison between and within the different materials. The inhibition zone was measured, and the level of statistical significance was set at ≤0.05.

## Results

**Figure 1:**
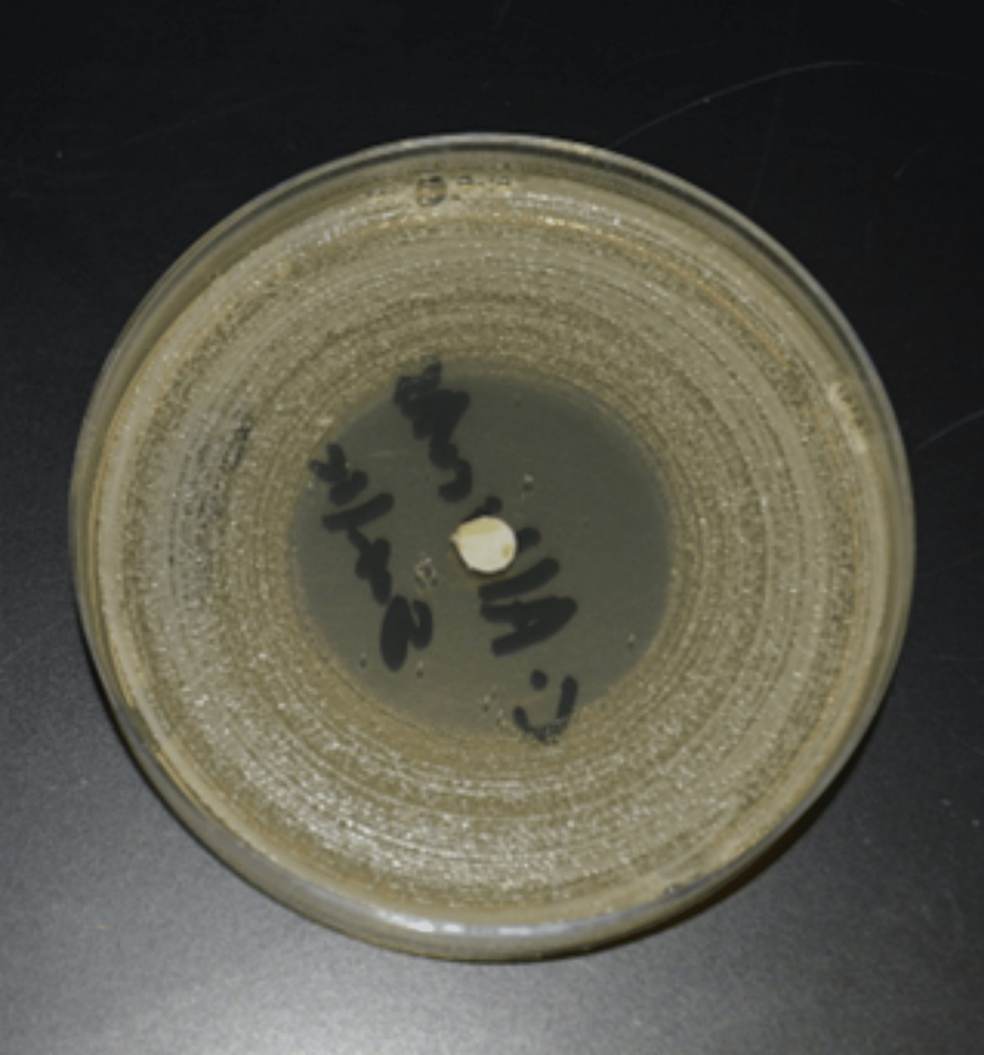
Inhibition zones of pure garlic extract

The diameter of inhibition zones has been measured along the vertical and horizontal axis. No inhibition zones were observed for the onion and lemon extracts used in this study whereas the garlic extract exhibited inhibition zones with altered sizes, as shown in Figure 1, with a mean and standard deviation of 4.89 ± 0.275. A highly statistically significant difference was observed between garlic and the other materials using one-way ANOVA (P = 0.000), as shown in Table 1, and between and within the materials using the chi-square test (P = 0.000), as shown in Table 2.

**Table 1:** Comparative differences between lemon extract, garlic, and onion using one-way ANOVA * Statistically significant level at P < 0.05. N: number of specimens; MCT: multiple comparison test (post hoc test).

#	N	Mean ± SD	P-value	95% confidence interval		MCT		
				Lower bound	Upper bound	Lemon	Garlic	Onion
Lemon	10	0.00 ± 0.000	0.000*	0.000	0.000	1.000	0.000	0.000
Garlic	10	4.89 ± 0.275		4.698	5.092	0.000	1.000	0.000
Onion	10	0.00 ± 0.000		0.000	0.000	1.000	0.000	1.000

**Table 2:** Comparative differences between and within the groups using the chi-square test * Statistically significant level at P < 0.05. D.F.: degree of freedom.

Variables		Comparison		Total	Chi-square	D.F.	P-value
		No inhibition	Inhibition				
Lemon	Count	10	0	10	30.000	2	0.000*
	% within group	100.0%	0.0%	100.0%			
Garlic	Count	0	10	10			
	% within group	0.0%	100.0%	100.0%			
Onion	Count	10	0	10			
	% within group	100.0%	0.0%	100.0%			

## Discussion

The present research was undertaken to evaluate the antifungal efficacy of pure garlic, onion, and lemon juice extracts on *C. albicans*. Where a positive correlation with statistically significant inhibition zones was highly noted with garlic but not with onion and lemon juice extracts.

Earlier research carried out by Watanabe in 1966 reported that garlic has a significant effect against pathogenic yeast and fungi [29]. He added that garlic has powerful natural antifungal properties that can have an effect not only on *Candida* but also aid to sustain healthy microbiomes in the gastrointestinal tract by abolishing harmful bacteria while sustaining healthy bacteria in place. Garlic stimulates the liver and colon, giving it a powerful additional effect on the body’s detoxification processes. Furthermore, garlic improves the function of the lymphatic system, by enhancing the body to liberate waste materials more competently.

Subsequent studies have also come to support the present finding, which is attributed to the ability of garlic (allicin) to inhibit succinate dehydrogenase, an enzyme complex that is crucial for the survival of most microorganisms [30,31]. The present results showed a highly significant inhibition zone when garlic was compared with the other different organic substances against *C. albicans*. Fresh garlic extract is more efficient than garlic powder extract as revealed by its morphological and inhibitory growth effects. Several other researchers have also experimented with components manufactured from plant extracts, which have also demonstrated favorable biological activity with an anti-fungal reaction. They revealed that the higher the concentration of garlic extract, the bigger the inhibition zone of *C. albicans*' growth [32-34]. Some other researchers have evaluated standard anti-fungal drug compounds against antifungal agents of plant origin [33].

The effect of allicin alone and in combination with fluconazole was investigated against *Candida* species and their synergistic effect was reviewed. The drug combination was proven to be efficient with acceptable anti-fungal properties [22]. In a recent study, Carreón-Delgado et al. stated that garlic peel extracts could be used as an antifungal agent too. Moreover, the application of selected garlic extracts as a preventive treatment presented a significant reduction in fungal growth after seven days of inoculation [35].

The antifungal properties of ajoene are well-verified, but its precise mechanisms of action are not yet understood. Numerous researchers stated that ajoene has a major impact on the growth inhibition of *C. albicans*, while others reported that aqueous garlic extract was more effective in inhibiting the growth of *C. albicans*. Garlic has shown apparent anti-*Candida* activity, with the power to halt the growth and spread of *C. albicans*. Supplementary to other antifungals, researchers assume that ajoene acts by disrupting the cell wall of the *Candida* yeast cells and in turn prevents them to function appropriately. There is also evidence that garlic can dispute *Candida* biofilms [36].

Popular around the world, onions are celebrated for their strong antibacterial, anti-parasitic, and less likely antifungal properties. In a study conducted by Ebrahimi et al. [37], it was shown that onion extracts had a more potent antibacterial effect against *Streptococcus mutans* than *Streptococcus sanguinis*. The antibacterial activity of red onions was more pronounced than yellow and green onions, respectively. As the concentration of the onions increased, the antibacterial activity also increased [37]. It was also reported that onions help the kidneys to flush excess fluids out of the body. It is highly beneficial for *Candida* sufferers who experience water retention. On the other hand, Genatrika et al. [38] reported that the gel from an extract of red onion showed significant antifungal activity. The antifungal activity of red onion occurred because it contained allicin. Similarly, it was cited that the essential oil of onion (*Allium cepa* L.) could inhibit the fungal growth of *C. albicans* [39]. However, our findings did not support the previous studies, as no inhibition zones were observed indicating the absence of antifungal efficacy against *C. albicans*.

In a study by Mathai et al. [40], lemon demonstrated antibacterial activities. When combinations of lemon extracts were tested against *Streptococcus mutans*, the lemon and garlic combination showed the greatest zone of inhibition than other organic materials in their study. In contradistinction to the present findings, the lemon juice extract did not show any antifungal activity against *C. albicans*. This could be attributed to the fact that 100% pure lemon juice extract was used in the present study. Hernawan et al. [24] carried out similar research with eight different concentrations of *Citrus limon* essential oil. *C albicans* did not grow on media with 100% essential oil treatment, but it grew on media with 50% essential oil treatment. They reported that the number of *C. albicans* colonies decreased when *Citrus limon* essential oil concentration decreased to less than 80%. Whereas Abdu et al. demonstrated that citrus lemon peel has significant antifungal activity against *C. albicans* [41]. This might shed some light on the present finding where pure lemon juice extract was used rather than lemon peel extract.

Nevertheless, lemon and lime juice accelerate the peristaltic action of the colon, allowing the muscles to simultaneously squeeze, thus pushing the waste out of the body. Hence, the efficiency of the digestive system is improved. Lemons and limes are excellent body-basifying agents, and they assist the body to bring back balance, which in turn helps it to operate more efficiently [42]. Citrus lemon peel juice has been commonly used as a substance mixed with warm water and utilized as a mouthwash and to relieve pain and inflammation due to infection and injuries in the oral cavity [43].

The limitation of the present study was basically the use of the traditional way only to extract fresh juices from the onion, garlic, and lemon material, and we did not use advanced extraction methods. The reason was to apply a method that could be easily applicable to medically compromised patients, clinically diagnosed with fungal infection (*C. albicans).*

## Conclusions

Fresh garlic extract was more efficient as revealed by its morphological and inhibitory growth effects with highly significant antifungal efficacy when compared to the onion and lemon juice extracts against *C. albicans*.

Further studies are needed using different concentrations and preparation modes of onion, lemon, and lime juice, in addition to the lemon and lime peel to confirm their antifungal efficacy in addition to their actual antimicrobial benefits.
